# Molecular Identification of Protozoan *Sarcocystis* in Different Types of Water Bodies in Lithuania

**DOI:** 10.3390/life13010051

**Published:** 2022-12-24

**Authors:** Agnė Baranauskaitė, Živilė Strazdaitė-Žielienė, Elena Servienė, Dalius Butkauskas, Petras Prakas

**Affiliations:** Nature Research Centre, Akademijos Str. 2, 08412 Vilnius, Lithuania

**Keywords:** environment, *Sarcocystis* species, molecular detection of parasites, water samples

## Abstract

Representatives of the genus *Sarcocystis* are unicellular parasites having a two-host life cycle and infecting mammals, birds, and reptiles. Until now, *Sarcocystis* spp. have been mainly investigated in definitive and intermediate hosts. Only a few studies have been conducted on the detection of *Sarcocystis* parasites in water samples. The aim of this research was to examine whether the prevalence of *Sarcocystis* spp. parasitizing farm animals varies in different types of water bodies. Water samples (*n* = 150) were collected from the entire territory of Lithuania, dividing water bodies into five groups (lakes, rivers, ponds/canals, swamps, and the inshore zone of the territorial Baltic Sea area). One-liter samples were filtered and subsequently analyzed using nested PCR. At least one of the analyzed *Sarcocystis* spp. (*S*. *arieticanis*, *S*. *bertrami*, *S*. *bovifelis*, *S*. *capracanis*, *S*. *cruzi*, *S*. *hirsuta*, *S*. *miescheriana*, and *S*. *tenella*) was determined in all examined samples from water bodies. No significant difference in *Sarcocystis* spp. prevalence between different types of water sources was detected. Our research proved that selecting appropriate primers is important for the accurate identification of parasites in samples collected from water bodies.

## 1. Introduction

Unicellular parasites of the genus *Sarcocystis* are characterized by an obligatory two-host life cycle. These parasites infect mammals, birds, and reptiles. Sarcocysts mostly develop in the muscle tissues of intermediate hosts; meanwhile, sporulation of oocysts occurs in the small intestine of the definitive host [1]. Four of more than 200 species belonging to this genus are zoonotic, *S*. *hominis*, *S*. *suihominis*, *S*. *heydorni*, and *S*. *nesbitti* [2]. However, few studies have been carried out to determine the prevalence of these species, as they are often misidentified during morphological or molecular examination [3].

To date, most research has been conducted on *Sarcocystis* spp. infecting economically important domestic animals. Farm animals can become infected through food or water contaminated with fecal sporocysts of *Sarcocystis* spp. It has been observed that *Sarcocystis* species transmitted through canids are more dangerous to farm animals, and acute infections can cause such symptoms as fever, weight loss, anemia, reduction in wool and milk yield, abortion, or even death [4,5,6,7].

Until now, *Sarcocystis* infection was mainly investigated by performing morphological or molecular analysis of animal carcasses. However, the use of natural environment studies by avoiding animal carcass-based research is increasing. Even so, only a few studies were conducted to test for *Sarcocystis* spp. in water [8,9,10]. The aim of our previous study was to assess different sample preparation and common PCR methodologies for identifying *Sarcocystis* species in water. The environmental water samples were collected from ponds (*n* = 49), lakes (*n* = 35), rivers (*n* = 18), canals (*n* = 10), and lagoons (*n* = 2). DNA of at least one of eight tested *Sarcocystis* species (*S*. *arieticanis*, *S*. *bertrami*, *S*. *bovifelis*, *S*. *capracanis*, *S*. *cruzi*, *S*. *hirsuta*, *S*. *miescheriana*, and *S*. *tenella*) was detected in 111 of 114 (97.4%) water samples using nested PCR targeting *cox1* gene [10]. However, *Sarcocystis* species occurrence rates in different areas and types of water bodies were not compared. Therefore, the aim of this study was to compare detection rates of different *Sarcocystis* species in five types of water bodies—lakes, rivers, canals/ponds, swamps, and the inshore zone of the Baltic Sea—using molecular methods.

## 2. Materials and Methods

### 2.1. Sample Collection

**Figure 1:**
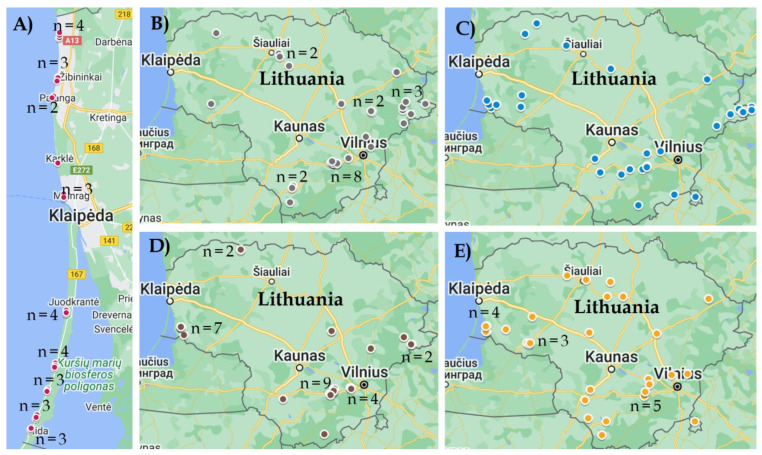
Water sampling sites in Lithuania in 2021. (**A**)—inshore zone of the Baltic Sea, (**B**)—lakes, (**C**)—rivers, (**D**)—swamps, (**E**)—ponds/canals.

Samples (*n* = 150) were collected from water bodies throughout the territory of Lithuania in the summer of 2021 (Figure 1). Water bodies were divided into five groups with an equal number of samples each—lakes (stagnant water), rivers (flowing water), canals and ponds (small water bodies that are usually close to pastures), swamps (acidic water) and the inshore zone of the Baltic Sea (saline water). Water samples were collected in sterile containers of 1 L capacity and transported in portable coolers with ice batteries. Until further processing, water samples were stored at +4 °C.

### 2.2. Preparation of Water Samples and Genomic DNA Extraction

First, the water sample was filtered through a metal sieve with 1 mm pores, then through Whatman™ Qualitative Filter Paper Grade 4 and finally filtered using MF-Millipore^®^ 5 μm pore membranes. Two milliliters of distilled water were used for membrane washing and collection of material, which was stored at +4 °C until further processing.

Genomic DNA (gDNA) was isolated from 200 μL of concentrated water samples using the GeneJET Genomic DNA Purification Kit (Thermo Fisher Scientific Baltics, Vilnius, Lithuania), according to the manufacturer’s recommendations. The resulting DNA samples were kept frozen at −20 °C until further analysis.

### 2.3. Nested PCR-Based Identification of Sarcocystis Parasites

During this work, samples from environmental water bodies were analyzed to distinguish the prevalence of different *Sarcocystis* spp. (*S*. *arieticanis*, *S*. *bertrami*, *S*. *bovifelis*, *S*. *capracanis*, *S*. *cruzi*, *S*. *hirsuta*, *S*. *miescheriana*, and *S*. *tenella*) using farm animals (cattle, sheep, goats, horses, and pigs) as intermediate hosts. Since the *cox1* gene is considered the most appropriate for the identification of selected *Sarcocystis* parasites [11,12,13], primers targeting this gene were selected [10]. It was observed that, having selected adequate PCR primer pairs, detection rates of farm animals infecting *Sarcocystis* spp. in water samples were associated with those observed in the muscles of the intermediate host [10,14,15,16,17]. However, in our earlier research, the prevalence of *S*. *bertrami, S*. *cruzi*, *S*. *miescheriana* and *S*. *tenella* identified in water samples was significantly lower [10]. Thus, 10 new primers (Table 1; highlighted in bold) were designed for the identification of these species. To detect *S*. *miescheriana* and *S*. *bertrami*, primers were redesigned to give shorter products, whereas to diagnose *S*. *cruzi* and *S*. *tenella*, different binding sites of primers were chosen.

During all PCR reactions, both positive (DNA extracted from sarcocysts of the corresponding *Sarcocystis* species) and negative (distilled water) controls were used. Primers were checked for cross reactions with other *Sarcocystis* species. The specificity of primer sets was confirmed. To check for possible contamination, distilled water and tap water were examined after the first batch, in the middle of our experiments and after the last batch. Based on the examination of distilled and tap water, PCRs were negative with all primers used in the study.

Preparation of PCR reaction mixtures and cycling conditions were as described previously [10]. The annealing temperatures were modified depending on the primers used (Table 1). Agarose gel electrophoresis was used to visualize PCR amplicons.

The selected PCR products were purified and directly sequenced as described previously [10]. Five positive samples of each species were used for sequencing, except for *S*. *hirsuta*, since only three samples were positive for this species. Four positive samples of the species *S*. *tenella*, *S*. *cruzi*, *S*. *bertrami* and *S*. *miescheriana* were additionally sequenced with the primers used in the previous study [10].

The editing of resolved sequences was performed manually with subsequent comparative BLAST analysis (http://blast.ncbi.nlm.nih.gov/, accessed on 10 October 2022). Differences in the prevalence of the identified *Sarcocystis* species were evaluated using the Chi-squared test. The *cox1* sequences of *Sarcocystis* species generated in the present study were deposited in the GenBank under the accession numbers OP681467–OP681524.

## 3. Results

### 3.1. Identification of Sarcocystis spp. Using Different PCR Primer Sets

GenBank accession numbers, length, and similarity of the obtained *cox1* sequences of *S*. *bovifelis*, *S*. *cruzi*, *S*. *hirsuta*, *S*. *arieticanis*, *S*. *tenella*, *S*. *capracanis*, *S*. *bertrami* and *S*. *miescheriana* are presented in Table 2. In no case did the obtained intraspecific and interspecific genetic differences overlap. Therefore, the primer sets used in this study were appropriate for the identification of *Sarcocystis* species in water samples examined.

**Figure 2:**
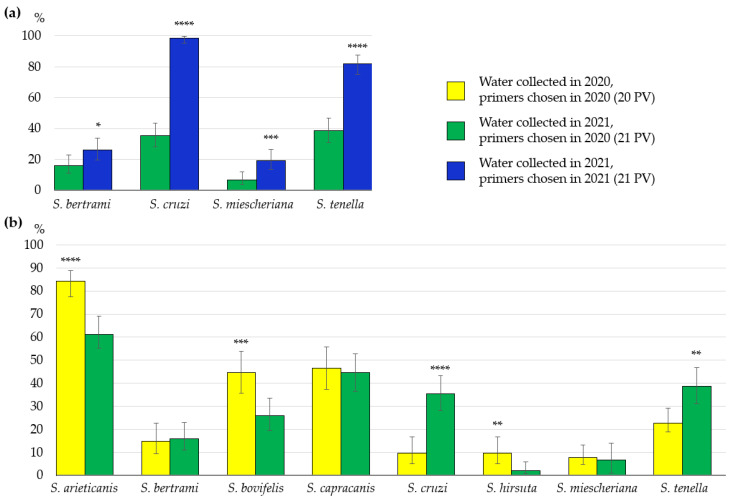
The detection frequency of examined *Sarcocystis* species in Lithuanian water bodies. (**a**) Comparison of detection rates of *Sarcocystis* spp. using different primer sets. (**b**) Comparison of detection rates of *Sarcocystis* spp. in 2020 [11] and 2021 (present study). * *p* < 0.05, ** *p* < 0.01, *** *p* < 0.001, **** *p* < 0.0001.

Four of eight investigated *Sarcocystis* species, *S*. *bertrami*, *S*. *cruzi*, *S*. *miescheriana* and *S*. *tenella*, were identified in the same water samples using two different primer combinations, the primer set chosen in our previous study (21 PV) [10] and the primer set selected in the current work (21 PS). The *Sarcocystis* parasite occurrence rate for the above-mentioned species was significantly higher (*p* < 0.05) using the primers selected in this study (21 PS) (Figure 2a). Depending on the primers used, the prevalence of *S*. *bertrami* was 16.0% and 26.0% (χ^2^ = 4.52, *p* < 0.05), whereas the prevalence of *S*. *miescheriana* accounted for 6.7% and 19.3% (χ^2^ = 10.64, *p* < 0.01). Most significant differences were identified when evaluating the primers tested for the detection of *S*. *cruzi* and *S*. *tenella*. The prevalence of *S*. *cruzi* was 35.3% and 98.7% (χ^2^ = 136.02, *p* < 0.00001) and the occurrence of *S*. *tenella* was 38.7% and 82.0% (χ^2^ = 58.85, *p* < 0.00001), using 21 PV and 21 PS, respectively.

The detection frequency of eight *Sarcocystis* species was compared in 150 water samples collected during the course of the present study (21 PV) and in 114 water samples collected throughout Lithuania in our previous investigation (20 PV) (Figure 2b). During both studies, *Sarcocystis* spp. were identified by the same technique (including same primer combinations). Statistically insignificant differences were observed in the cases of *S*. *bertrami* (16.0% vs. 14.9%, χ^2^ = 0.06, *p* > 0.05), *S*. *capracanis* (44.7% vs. 46.5%, χ^2^ = 0.09, *p* < 0.05) and *S*. *miescheriana* (6.7% vs. 7.9%, χ^2^ = 0.15, *p* < 0.05). Significantly higher occurrence rates of *S*. *arieticanis* (84.2% vs. 61.3%, χ^2^ = 16.54, *p* < 0.001), *S*. *bovifelis* (44.7% vs. 26.0%, χ^2^ = 10.12, *p* < 0.01) and *S*. *hirsuta* (9.6% vs. 2.0%, χ^2^ = 7.55, *p* < 0.01) were calculated in previously collected water samples (20 PV), whereas significantly higher detection rates of *S*. *tenella* (38.7% vs. 22.8%, χ^2^ = 7.51, *p* < 0.01) and *S*. *cruzi* (35.3% vs. 9.6%, χ^2^ = 23.27, *p* < 0.001) were established in water samples obtained during the current work (21 PV).

### 3.2. Sarcocystis spp. Occurrence Rates in Different Types of Water Bodies

**Figure 3:**
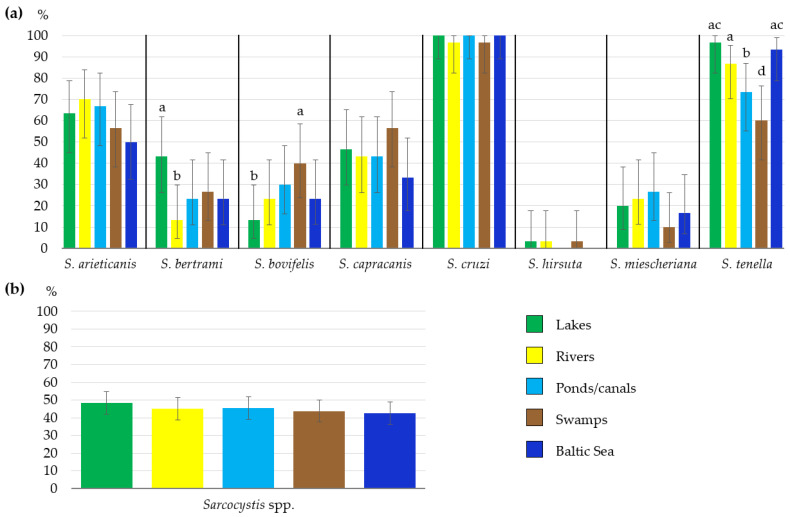
Identification of *Sarcocystis* species in different water bodies. (**a**) The detection rate of eight analyzed *Sarcocystis* species in different types of Lithuanian water bodies. (**b**) The overall frequency of *Sarcocystis* spp. in different water bodies. It was calculated by summing up all PCR-positive samples and dividing them by the total number of samples tested. a > b and c > d (*p* < 0.05).

The detection rate of *Sarcocystis* species examined was compared in five types of water bodies—lakes, rivers, ponds/canals, swamps, and the inshore zone of the Baltic Sea (Figure 3a). The comparison showed a significantly higher (χ^2^ = 6.65, *p* < 0.01) detection rate of *S*. *bertrami* in lakes (43.3%) than that in rivers (13.3%) and a significantly higher (χ^2^ = 5.45, *p* < 0.05) detection rate of *S*. *bovifelis* in swamps (40.0%) than that in lakes (13.3%). In the case of *S*. *tenella*, significantly higher detection rates were calculated in lakes (96.7%) than those in swamps (60.0%) (χ^2^ = 11.88, *p* < 0.001) and ponds/canals (73.3%) (χ^2^ = 6.41, *p* < 0.05); moreover, they were higher in the inshore zone of the Baltic Sea (93.3%) than the rates in swamps (χ^2^ = 9.32, *p* < 0.01) and ponds/canals (χ^2^ = 4.32, *p* < 0.05), and finally, they were higher (86.7%) in rivers as compared to those in swamps (χ^2^ = 5.45, *p* < 0.05). The overall frequency of *Sarcocystis* species (calculated by summing up all PCR-positive samples and dividing them by the total number of samples tested) varied depending on water type: from 102 (42.5%) positive cases in the Baltic Sea to 116 (48.3%) positive cases in lakes (Figure 3b). However, the differences observed in occurrence rates of *Sarcocystis* spp. in five types of water bodies were insignificant (χ^2^ = 1.85, df = 4, *p* > 0.05). In summary, the overall prevalence of *Sarcocystis* species did not depend on the type of water body.

### 3.3. Distribution of Sarcocystis spp. in Water Samples

Summarizing the results, at least one *Sarcocystis* species was identified in all 150 examined samples. The number of *Sarcocystis* species per individual sample was estimated by combining data obtained in the analysis using different primer sets (21 PV and 21 PS). Single species was identified only in three cases (2.0%). The detection of two (18.7%), three (21.3%), four (32.0%) and five (19.3%) *Sarcocystis* species per sample was more frequent. Finally, six and seven species of *Sarcocystis* in one water sample were identified in nine cases (6.0%) and one (0.7%) case, respectively.

In the present work, the lowest *Sarcocystis* species detection rate in water samples analyzed was established for *S*. *hirsuta*, and was equal to 2%. The identification rates of *S*. *bertrami* (26.7%), *S*. *bovifelis* (26.0%) and *S*. *miescheriana* (19.3%) did not exceed 30%. Moderate detection frequency was estimated for *S*. *capracanis* (44.7%) and *S*. *arieticanis* (61.3%), whereas the highest prevalence was revealed for *S*. *tenella* (89.3%) and *S*. *cruzi* (99.3%).

## 4. Discussion

Based on nested PCR, we identified eight *Sarcocystis* species (*S*. *arieticanis*, *S*. *bertrami*, *S*. *bovifelis*, *S*. *capracanis*, *S*. *cruzi*, *S*. *hirsuta*, *S*. *miescheriana*, and *S*. *tenella*) in different types of water bodies (Table 2, Figure 3). Cattle are intermediate hosts for the first three *Sarcocystis* species tested, *S*. *arieticanis* and *S*. *tenella* use sheep as their intermediate hosts, while goats, horses and pigs/wild boar are hosts of *S*. *capracanis*, *S*. *bertrami*, and *S*. *miescheriana*, respectively [5]. Based on current knowledge, the European bison (*Bison bonasus*) can be an alternative host for *Sarcocystis* species parasitizing cattle [18]. The sarcocysts of such species as *S*. *arieticanis*, *S*. *tenella* and *S*. *capracanis* can be found in muscle tissues of European mouflon (*Ovis aries musimon*) [19]. Nevertheless, in the areas under investigation, the mentioned wild animals are rare [20]. Other wild ungulate species that could be intermediate hosts for the tested *Sarcocystis* species are not free-ranging in Lithuania.

To date, most studies have been conducted on the prevalence of protozoan infection in drinking water treatment facilities [21,22,23,24], whereas only a few studies have investigated the prevalence of parasitic protozoa in environmental water sources, such as rivers [25,26], reservoirs [27], lakes [28] or private wells [29]. Typically, studies cover small regions, basins of a particular river or several nearby villages where an outbreak of parasitic protozoa was identified. The number of studies on other parasitic protozoa is much smaller; for example, only three studies were devoted to the identification of *Sarcocystis* spp. in water bodies [8,9,10]. The current study is the first attempt to compare the prevalence of eight *Sarcocystis* species in different types of water bodies. It is noteworthy that equal numbers of samples representing five groups of water bodies (lakes, rivers, ponds/canals, swamps, and the Baltic Sea) were collected throughout the entire territory of the country. The majority of other investigations of parasitic protozoa in water samples were limited to small geographic regions or the location of infection outbreaks [22,27,28,29]. During the present study, it was found that overall detection of the analyzed *Sarcocystis* species did not depend on the type of water body (Figure 3). The number of positive cases was very similar in all types of water and varied from 102 to 116 positive cases. As a result, it can be assumed that environmental conditions and the location of water bodies had no effect on the prevalence of *Sarcocystis* parasites in different water sources. However, insignificant differences were noticed when comparing the distribution of eight species in different types of water bodies. Distribution of individual *Sarcocystis* species may be determined by the abundance of final hosts in a particular area and different characteristics of water bodies, such as water salinity, acidity, or flow turbidity.

Our current research showed that *Sarcocystis* spp. DNA detection depended on the primer combinations used (Figure 2). The use of different primer pairs for identification of *S*. *bertrami*, *S*. *miescheriana*, *S*. *cruzi*, and *S*. *tenella* revealed statistically significant differences. As compared to the prevalence of these four *Sarcocystis* species obtained using primers selected in the previous study (21 PV) [10], a significantly higher (*p* < 0.05) prevalence was determined when the primers selected in this study (21 PS) were applied to PCR. Previous studies also showed that amplification success of *cox1* fragments of *Sarcocystis* spp. using ungulates as their intermediate hosts depended on the chosen PCR primers [30,31,32]. It is assumed that some *Sarcocystis* species exhibit high interspecific genetic variability [17,33]. However, intraspecific genetic variation of *Sarcocystis* spp. is poorly studied [34,35,36], and selection of suitable primers has been one of the challenges to the diagnosis of *Sarcocystis* species thus far.

Due to the large morphological and genetic differences between genera or even species of parasitic protozoa, universal methods for identification of these parasites in various environmental samples have not been developed yet. The concentration of many protozoa species in water samples is relatively low [37]; therefore, detection requires very sensitive techniques, such as molecular methods. While some protozoa are undetectable in water samples under a microscope [38], molecular methods for the identification of parasitic protozoa are not fully developed [27,37]. It should be noted that molecular-based techniques are not standardized for the diagnosis of *Sarcocystis* and other parasitic protozoa in water samples [10,37]. Therefore, a wide variety of methodologies, e.g., conventional PCR and derivatives, DNA hybridization, loop-mediated isothermal amplification (LAMP), or quantitative PCR, are used [10,27,39,40]. During environmental sample testing, discrimination between DNA from a living cell versus DNA from a dead one might be considered impossible. Consequently, the prevalence of parasites in the samples collected can be overestimated [37]. Oocysts or sporocysts of protozoan *Sarcocystis* parasites are also known to be resistant to various environmental conditions (freezing, low humidity, high temperatures, etc.) and can remain viable in nature for months [5,41]. However, to amplify DNA only from viable sporocysts, water samples can be treated with dyes that penetrate only membrane-damaged cells. After dye has entered the cell, it is covalently cross-linked to DNA. Consequently, PCR amplification is strongly inhibited [42]. To date, fluorescent dyes, such as ethidium monoazide (EMA), propidium monoazide (PMA), ethidium bromide (EB), and propidium iodide (PI), have been successfully used in protozoan studies [43]. Accordingly, further research of *Sarcocystis* spp. in water samples could focus on additional testing, such as measuring the sensitivity of sporocyst recovery, a spike-and-recovery experiment (controlled contamination of water samples with purified sporocysts) or adaptation of quantitative PCR. However, during the previous studies on animal carcasses, a high prevalence of *Sarcocystis* spp. infection was determined in some intermediate hosts [14,15,20]. As much as 100% of cattle and sheep bred in Lithuania were found to be infected with *Sarcocystis* parasites [14,15]. Meanwhile, the prevalence of infection in horses and pigs accounted for 47.2% and 40.2%, respectively [14]. Since it is known that animals can become infected with these parasites through food and water, there seem to be large amounts of viable and infective sporocysts in the environment.

## 5. Conclusions

The present study is the first attempt to compare detection rates of *Sarcocystis* parasites using farm animals as their intermediate hosts in five different types of water bodies (lakes, rivers, ponds/canals, swamps, and the inshore zone of the Baltic Sea). The prevalence of the analyzed *Sarcocystis* species did not vary significantly between the examined water source groups. The environmental conditions of water bodies do not affect the prevalence of *Sarcocystis* parasites. Based on the nested PCR, eight *Sarcocystis* species were identified—*S. hirsuta* (2.0%), *S. miescheriana* (19.3%), *S. bovifelis* (26.0%), *S. bertrami* (26.7%), *S. capracanis* (44.7%), *S. arieticanis* (61.3%), *S. tenella* (89.3%) and *S. cruzi* (99.3%). Further, it was established that the detection frequency of *Sarcocystis* species in water samples depended on the combinations of selected primers. The present study showed that in general, *Sarcocystis* parasites were widespread in water bodies and could easily infect livestock.

## Figures and Tables

**Table 1:** List of oligonucleotides used for nested PCR reaction.

Species		Primers				Ta, °C	ExT, s	ProS, bp
	PCR Round	Primer Origin	Name	Orientation	Sequence (5′–3′)			
*S. bovifelis*	1st	PV	V2bo1	Forward	AACTTCCTAGGTACAGCGGTATTCG	60	40	556
			V2bo2	Reverse	TGAACAGCAGTACGAAGGCAAC			
	2nd		V2bo3	Forward	ATATTTACCGGTGCCGTACTTATGTT	60	30	410
			V2bo4	Reverse	GCCACATCATTGGTGCTTAGTCT			
*S. cruzi*	1st	PV	V2cr1	Forward	TACAATGTGCTGTTTACGCTCCA	57	50	776
			V2cr2	Reverse	GCAATCATGATAGTTACGGCAGA			
	2nd		V2cr3	Forward	ACCATCCTGTTCTGTGGTGCTATG	65	30	298
			V2cr4	Reverse	AAACTACTTTACTGCCTACGGTACTC			
	1st	PS	V2cr1	Forward	TACAATGTGCTGTTTACGCTCCA	63	55	777
			**V2cr2a**	Reverse	CAATCATGATAGTTACGGCAGAGA			
	2nd		**V2cr3c**	Forward	TCCAAGTACACGGCATTATTTACC	59	30	268
			V2cr4	Reverse	AAACTACTTTACTGCCTACGGTACTC			
*S. hirsuta*	1st	PV	V2hi5	Forward	TATGTTGGTTCTGCCGAAGTCAT	60	45	686
			V2hi6	Reverse	GGTATGGCAATCATTATGGTTACAG			
	2nd		V2hi7	Forward	GCACCGTAATATTTCAGGGATGT	60	30	299
			V2hi8	Reverse	AACCTGCTTGCCGGAGTAAGTA			
*S. arieticanis*	1st	PV	V2arie1	Forward	CTCTTTGCCGTAGATTCGCTAGTTA	63	55	884
			V2arie2	Reverse	CAAAGATCGGTAGATATCCAATGC			
	2nd		V2arie3	Forward	TAGTTCTTGGCCTGGCTATTCTT	59	30	371
			V2arie4	Reverse	CTGACCTCCAAAAACTGGCTTAC			
*S. tenella*	1st	PV	V2te1	Forward	GAGCGGTGAACTTCTTAGGAACC	60	40	537
			V2te2	Reverse	CCCAATAATCCGCTGTTAACGTA			
	2nd		V2te3b	Forward	ATTGTAATGCTCCTCGACGATATG	57	30	314
			V2te4	Reverse	ATAGTCACGGCAGAGAAGTAGGAC			
	1st	PS	V2te1	Forward	GAGCGGTGAACTTCTTAGGAACC	60	40	537
			V2te2	Reverse	CCCAATAATCCGCTGTTAACGTA			
	2nd		**V2te3c**	Forward	ATGTTGATCATAACCATACCGATATTC	61	30	348
			V2te4	Reverse	ATAGTCACGGCAGAGAAGTAGGAC			
*S. capracanis*	1st	PV	VocaF	Forward	GTAAACTTCCTGGGTACTGTGCTGT	60	40	531
			VocaR1	Reverse	CCAGTAATCCGCTGTCAAGATAC			
	2nd		V2cap3	Forward	ATACCGATCTTTACGGGAGCAGTA	63	30	330
			V2cap4	Reverse	GGTCACCGCAGAGAAGTACGAT			
*S. bertrami*	1st	PV	V2ber1	Forward	GTATGAACTGTCAACGGATGGAGTA	58	60	883
			V2ber2	Reverse	AGAAGCCATGTTCGTGACTACC			
	2nd		V2ber3	Forward	GTACTACCTCCTTCCAGTCGGTTC	57	40	600
			V2ber4	Reverse	CGGGTATCCACTTCAAGTCCAG			
	1st	PS	V2ber3	Forward	GTACTACCTCCTTCCAGTCGGTTC	58	45	605
			**V2ber6**	Reverse	ACGACCGGGTATCCACTTCA			
	2nd		**V2ber7**	Forward	CCCCACTCAGTACGAACTCC	59	30	381
			**V2ber8**	Reverse	ACTGCGATATAACTCCAAAACCA			
*S. miescheriana*	1st	PV	V2mie1	Forward	TGCTGCGGTATGAACTATCTACCT	61	60	922
			V2mie2	Reverse	GCCCAGAGATCCAAATCCAG			
	2nd		V2mie3	Forward	CTTGGTTCAACGTTACTCCTCCA	61	30	474
			V2mie4	Reverse	CTTCGATCCAGCTGAACTAAAGC			
	1st	PS	V2mie3	Forward	CTTGGTTCAACGTTACTCCTCCA	58	50	701
			V2mie2	Reverse	GCCCAGAGATCCAAATCCAG			
	2nd		**V2mie5**	Forward	TCCTCGGTATTAGCAGCGTACTG	55	30	358
			**V2mie6**	Reverse	ATTGAAGGGCCACCAAACAC			

PV are primer pairs selected in the previous study [10], PS are primer combinations selected in the present study. Ta is annealing temperature, Ext is extension time, ProS is product size. Primers designed in the present study are in boldface.

**Table 2:** Nested PCR-based identification of different *Sarcocystis* species.

Species	Assigned No. in GenBank (Length, bp)	Position of *cox1* Fragment Corresponding to *S. gracilis* MN339303	Sequence Similarity, %	
			Comparison of Acquired Sequences vs. the Same Species Accessible in GenBank	Comparison of Acquired Sequences vs. Greatly Related Species
*S. bovifelis*	OP681482–OP681486 (361)	594–954	99.5–100	*S*. *bovini* 93.1–94.5
*S. cruzi*	OP681492–OP681501 (248, 218)	493–741 ^a^, 523–741 ^b^	95.4–100	*S*. *levinei* 89.9–90.8
*S. hirsuta*	OP681502–OP681504 (254)	490–743	97.6–100	*S*. *buffalonis* 92.4–93.2
*S. arieticanis*	OP681467–OP681471 (325)	430–754	92.6 *–99.4	*S*. *hircicanis* 86.5–87.4
*S. tenella*	OP681515–OP681524 (263, 296)	607–869 ^c^, 574–869 ^d^	96.3–100	*S*. *capracanis* 91.3–93.2
*S. capracanis*	OP681487–OP681491 (284)	586–869	96.8–99.7	*S*. *tenella* 90.4–92.9
*S. bertrami*	OP681472–OP681481 (554, 336)	294–847 ^e^, 376–711 ^f^	96.4–99.8	*S*. *matsuoae* 77.9–79.7
*S. miescheriana*	OP681505–OP681514 (428, 315)	308–739 ^g^, 448–765 ^h^	92.4 **–99.4	*S*. *rangiferi* 76.8–80.4

Primers used for PCR: ^a^ V2cr1/V2cr2 and V2cr3/V2cr4, ^b^ V2cr1/V2cr2a and V2cr3c/V2cr4, ^c^ V2te1/V2te2 and V2te3b/V2te4, ^d^ V2te1/V2te2 and V2te3c/V2te4, ^e^ V2ber1/V2ber2 and V2ber3/V2ber4, ^f^ V2ber3/V2ber6 and V2ber7/V2ber8, ^g^ V2mie1/V2mie2 and V2mie3/V2mie4, ^h^ V2mie3/V2mie2 and V2mie5/V2mie6. * 98.5–100% similarity with most isolates of *S*. *arieticanis* obtained from Europe, and 92.6–93.5% similarity with *S*. *arieticanis* isolated from Egypt; ** 96.8–100% similarity with European isolates of *S*. *miescheriana*, and 92.4–95.3% similarity with Asian *S*. *miescheriana* isolates.

## Data Availability

Data supporting the conclusions of this article are included in the article. The sequences generated in the present study were submitted to the GenBank database under Accession Numbers OP681467–OP681524.
